# Socioeconomic Characteristics of Communities With Primary Care Practices With Nurse Practitioners

**DOI:** 10.1001/jamanetworkopen.2024.62360

**Published:** 2025-02-28

**Authors:** Monica O’Reilly-Jacob, Kyle G. Featherston, Hilary Barnes, Ying Xue, Lusine Poghosyan

**Affiliations:** 1 School of Nursing, Columbia University, New York, New York; 2 School of Nursing, Widener University, Chester, Pennsylvania; 3 School of Nursing, University of Rochester, Rochester, New York

## Abstract

**Importance:**

Given the disparities in access to primary care and the growing nurse practitioner (NP) workforce, it is important to understand the distribution of primary care practices with NPs across communities of varying socioeconomic characteristics.

**Objective:**

To compare the socioeconomic characteristics of communities that have primary care practices with or without NPs.

**Design, Setting, and Participants:**

This secondary, cross-sectional analysis of 79 743 primary care practices used 4 merged data sources: the IQVIA 2023 OneKey database, the 2020 US biennial Census, the 2017-2022 American Community Survey, and the 2021 Area Deprivation Index (ADI). US Census Tracts and Divisions from 2023 were used to examine differences in socioeconomic characteristics across communities.

**Exposure:**

Primary care practices with NPs vs without NPs.

**Main Outcomes and Measures:**

Socioeconomic characteristics of Census Tracts and Block Groups, including racial and ethnic composition, median household income, percentage below the federal poverty level, educational attainment, and the ADI (an ordinal percentile ranking of Census Block Groups from 1 to 100, with 1 being least disadvantaged and 100 being most disadvantaged).

**Results:**

Of 79 743 primary care practices, 42 601 (53.4%) employed NPs in 2023. Practices with NPs, compared with those without, were significantly more likely to be in communities classified as low income (23.3% vs 17.0%; *P* < .001) and rural (11.9% vs 5.5%; *P* < .001). On average, these communities had a higher proportion of the population living below the federal poverty level (14.4% [95% CI, 14.3%-14.5%] vs 12.8% [95% CI, 12.7%-12.9%]; *P* < .001) and without a high school diploma (19.8% [95% CI, 19.7%-19.9%] vs 18.5% [95% CI, 18.4%-18.6%]; *P* < .001). Communities with practices with NPs also had significantly higher mean ADI percentiles than communities with practices without NPs (53.3% [95% CI, 53.1%-53.6%] vs 42.5% [95% CI, 42.2%-42.7%]; *P* < .001). As the number of primary care practices decreased in disadvantaged areas, the proportion of practices with NPs increased. In most US Census Divisions, there were more primary care practices with NPs than without, a difference that was marked in low-income communities.

**Conclusions and Relevance:**

This cross-sectional study of primary care practices in the US found that primary care practices with NPs, compared with those without NPs, were more likely to be located in communities with lower income and educational attainment and greater levels of overall socioeconomic disadvantage. This finding suggests that NPs are key to ensuring access to primary care in communities with socioeconomic disadvantage.

## Key Points

**Question:**

Are primary care practices with nurse practitioners (NPs) more likely than practices without NPs to be located in communities with low socioeconomic status?

**Findings:**

This cross-sectional study of 79 743 primary care practices in the US found that practices with NPs, compared with those without NPs, were more likely to be located in communities with lower income and educational attainment and greater levels of overall socioeconomic disadvantage. Practices with NPs predominated in areas with the highest need for but the lowest supply of primary care practices.

**Meaning:**

This study suggests that NPs are key to ensuring access to primary care in communities of greater socioeconomic disadvantage.

## Teaser

This cross-sectional study compares the socioeconomic characteristics of communities that have primary care practices with or without nurse practitioners (NPs).

## Introduction

The nurse practitioner (NP) workforce is rapidly expanding, reaching 385 000 NPs in 2023, with 45% growth projected between 2022 and 2032.^1,2^ Primary care practices increasingly rely on NPs for care delivery to increase access to care.^3^ However, to date, little is known about the nationwide distribution of primary care practices employing NPs and their predominance in particular types of communities (ie, rural, low socioeconomic status, and racially and ethnically diverse). As the US confronts the ongoing challenge of eliminating disparities in access to care,^4,5,6^ it is important to better understand the characteristics of communities where primary care practices employ NPs.

Primary care is critical to improving population health,^7^ especially for people living in communities with socioeconomic disadvantage who are at high risk for frequent emergency department visits, preventable hospitalizations, and hospital readmissions.^8,9,10,11^ However, accessing primary care in communities with socioeconomic disadvantage is difficult, largely due to a maldistribution of primary care clinicians that cluster in resourced areas.^4,12^ Nurse practitioners are an important resource for primary care delivery, and may contribute to improved access to primary care in underserved areas.^13,14^ However, accurately counting primary care NPs and understanding where they are located is complicated by a lack of basic information on NP specialty in commonly used data sources (eg, administrative claims).^15,16^ For this reason, little is known about the types of communities where primary care practices with NPs are located, particularly communities of socioeconomic disadvantage. It is important to understand the socioeconomic characteristics of the communities served by NPs to inform strategies to maintain a robust and well-distributed primary care workforce across the nation.

We addressed this gap by using a unique and reliable data source of primary care practices and community characteristics, including the Area Deprivation Index (ADI). With this novel dataset we sought to describe differences in the socioeconomic characteristics of communities and assess whether primary care practices with NPs were more likely than primary care practices without NPs to be located in communities with socioeconomic disadvantage.

## Methods

To explore the differences in socioeconomic characteristics of communities where NPs provide primary care and where they do not, we merged primary care practice data from IQVIA’s 2023 OneKey database, the 2020 US biennial Census data, the 2017-2022 American Community Survey (ACS), and the 2021 ADI. Institutional review board approval was waived by Columbia University as the data used in this study contained no identifiable information, ensuring compliance with institutional policies and ethical guidelines for research involving nonidentifiable data. The report of this cross-sectional study follows the Strengthening the Reporting of Observational Studies in Epidemiology (STROBE) reporting guideline.

### Data Sources

#### IQVIA’s OneKey Database

We created a dataset of primary care practices by extracting the list of all primary care practices in the US in 2023 from IQVIA’s proprietary OneKey database. We used accepted criteria to develop a multistep approach to identify primary care practices.^3^ We defined primary care practices as (1) all independent physician practices where the physician held a primary care specialty (ie, family medicine, general practice, internal medicine, preventive medicine, or geriatrics), or (2) medical groups with facility specialty identified as primary care, or (3) medical groups where the facility specialty was not primary care but 50% or more of the physicians held a primary care specialty. The final analytic sample was limited to information only about NPs.

#### US Census Bureau

We obtained data from the 2020 biennial US Census and used a range of geographic units for our analysis. We used Census Tracts to examine differences in socioeconomic characteristics and racial and ethnic population statistics across local communities. The racial and ethnic group populations as determined by the US Census Bureau were obtained to describe the racial and ethnic populations of neighborhoods that primary care practices served. Census Tracts are small (ie, mean, 4000 individuals [range, 1200-8000 individuals]), relatively permanent statistical subdivisions of a county. We used Census Block Group to examine differences in overall socioeconomic disadvantage and Census Division to examine variation across regions.

The Census Bureau also conducts the ACS, an ongoing survey covering a broad range of topics about the social, economic, housing, and demographic characteristics of the US population.^17^ We used the 2017-2022 ACS 5-year estimates, the most recent data available, to obtain data on the characteristics of Census Tracts, including median household income, percentage below the federal poverty level, and level of educational attainment.

#### Area Deprivation Index

We used the 2021 ADI as a measure of the overall socioeconomic disadvantage of communities.^18^ The ADI, a composite of socioeconomic indicators in the ACS (eg, income, educational level, employment, and housing quality), is an ordinal percentile ranking of Census Block Groups from 1 to 100, with 1 being least disadvantaged and 100 being most disadvantaged.^18^

### Variables

For each practice in the primary care practice dataset, we acquired the number of NPs in the practice and the 9-digit zip code. Using the zip code, we merged the practice data with the 9-digit 2021 ADI file, which contains both the 9-digit zip code and a 12-digit geographic identifier (ie, Federal Information Processing Series code). The geographic identifier was then used to merge with a Census Tract’s racial and ethnic population statistics from the 2020 biennial Census and the socioeconomic data from the ACS.

#### Low-Income Status of the Census Tract

Census Tracts were categorized as low income if they were below the 20th percentile of household median income across all Census Tracts. We also conducted a sensitivity analysis using alternative definitions of *low income* (ie, tracts in the lowest quartile and the lowest decile of income, tracts with a median income of 80% of the national median, tracts with 20% or more of the population below the federal poverty level, and tracts with either the lowest quartile of income or 80% below the federal poverty level).

#### Rurality

Rural-Urban Commuting Area (RUCA) codes were obtained from the US Department of Agriculture Economic Research Service website for each Census Tract.^19^ The most recent (2010) data were used. Tracts with primary RUCA codes of 1 to 3 were considered metropolitan, tracts with RUCA codes between 4 and 6 were considered micropolitan, and tracts with RUCA codes greater than 6 were considered rural.

### Statistical Analysis

We tested for the significance of differences between practices with and practices without NPs using unequal variance *t* tests for continuous variables and χ^2^ tests for categorical variables. In addition to examining differences across the communities (ie, Census Tracts) where the practice was located, we also evaluated the geographic variation across regions (ie, Census Division). This also allowed us to investigate whether differences in local communities (eg, community income levels) also varied across larger geographic regions. In addition, we conducted a sensitivity analysis using alternative definitions of *low income* to test whether other definitions would produce similar results. Statistical analysis was performed with R statistical software, version 4.3.1 (R Project for Statistical Computing). All *P* values were from 2-sided tests and results were deemed statistically significant at *P* < .05.

## Results

We identified 79 743 primary care practices in the IQVIA 2023 OneKey database, of which 42 601 (53.4%) employed at least 1 NP. We linked 77 180 primary care practices (96.8%) to a Census Block Group and Tract. The number of NPs across practices ranged from 1 to 292, with a mean (SD) of 2.6 (3.4) (95% CI, 2.5-2.6) per practice. Table 1 displays the characteristics of the communities where primary care practices with or without NPs were located, and Table 2 shows the socioeconomic status of those communities using a range of definitions of low-income Census Tract. Practices with NPs, compared with those without, were significantly more likely to be in communities classified as low income (23.3% vs 17.0%; *P* < .001), as well as rural (11.9% vs 5.5%; *P* < .001). On average, communities where primary care practices with NPs were located had a higher proportion of the population living below the federal poverty level (14.4% [95% CI, 14.3%-14.5%] vs 12.8% [95% CI, 12.7%-12.9%]; *P* <.001]) and without a high school diploma (19.8% [95% CI, 19.7%-19.9%] vs 18.5% [95% CI, 18.4%-18.6%]; *P* < .001). We also found the median household income in communities with practices with NPs had a mean more than $10 000 lower ($74 467 [95% CI, $74 101-$74 772]) than in communities with practices without NPs ($85 621 [95% CI, $85 191-$86 050]). In terms of racial and ethnic composition, compared with practices without NPs, those with NPs served communities with larger non-Hispanic White populations (60.4% vs 56.5%; mean difference, 3.9% [95% CI, 3.3%-4.3%]) and larger Black or African American populations (12.2% vs 11.2%; mean difference, 1.0 [95% CI, 0.8%-1.2%]) but smaller Hispanic or Latino populations (17.0% vs 18.8%; mean difference, –1.8% [95% CI, −1.5% to −2.1%]) and smaller Asian populations (5.0% vs 8.2%; mean difference, –3.2% [95% CI, −3.1% to −3.4%]).

**Table 1.** Characteristics of Communities With Primary Care Practices With or Without NPs^a^

Characteristic	Practices with NPs	Practices without NPs
Population below the federal poverty level in Census Tract, %^b^		
Mean (SD)	14.4 (10.8)	12.8 (10.3)
Median (IQR)	11.2 (0-24.1)	10.0 (0-21.8)
Educational attainment of population in Census Tract, %^c^		
Bachelors degree or higher		
Mean (SD)	33.2 (19.1)	39.1 (20.8)
Median (IQR)	28.9 (1.6-56.1)	36.1 (3.8-68.4)
No high school		
Mean (SD)	19.8 (9.5)	18.5 (10.0)
Median (IQR)	18.5 (7.3-29.7)	17.1 (5.0-29.0)
Median household income of Census Tract, $^d^		
Mean (SD)	74 437 (35 304)	85 621 (42 225)
Median (IQR)	66 887 (27 268-106 504)	76 094 (26 583-125 605)
ADI of Census Block Group (national percentile)^e^		
Mean (SD)	53.3 (27.2)	42.5 (28.1)
Median (IQR)	54 (9-99)	39 (0-86)
Rurality of Census Tract, % (No.)^f^		
Rural	11.9 (5083)	5.5 (2049)
Micropolitan	11.3 (4811)	6.8 (2478)
Metropolitan	76.8 (32 690)	87.8 (37 121)

Abbreviations: ADI, Area Deprivation Index; NPs, nurse practitioners.

^a^ A small number of Census Tracts (2%-3%) were missing data, resulting in modestly different sample sizes for each analysis. All community characteristics between mean values of practices with NPs and mean values of practices without NPs were significantly different (*P* < .001), based on unequal variance *t* tests for continuous variables and χ^2^ tests for categorical variables.

^b^ Based on 76 193 practices (41 258 with NP) with poverty data.

^c^ Based on 76 237 practices (41 281 with NP) with educational data.

^d^ Based on 75 950 practices (41 137 with NP) with median income data (2022 dollar amount based on the 2017-2022 American Community Survey data).

^e^ Based on 74 707 practices (40 382 with NP) with ADI data.

^f^ Based on 79 705 practices (42 584 with NP) with rurality data.

**Table 2.** Sensitivity Analysis of Primary Care Practices With or Without NPs, Using Alternative Definitions of Low-Income Census Tracts^a^

Definition	Practices, No./total No. (%)		
	Without NPs	With NPs	Total
Lowest quintile	5932/34 813 (17.0)	9583/41 137 (23.3)	15 515/75 950 (20.4)
Lowest quartile	7447/34 813 (21.4)	12 013/41 137 (29.2)	19 460/75 950 (25.6)
Lowest decile	2753/34 813 (7.9)	4405/41 137 (10.7)	7158/75 950 (9.4)
80% of Median income	9251/34 813 (26.6)	14 612/41 137 (35.5)	23 863/75 950 (31.4)
>20% Below federal poverty level^b^	6592/34 935 (18.9)	9771/41 258 (23.7)	16 363/76 193 (21.5)
>20% Below federal poverty level or lowest quartile	9183/34 813 (26.4)	13 997/41 137 (34.0)	23 180/75 950 (30.5)

Abbreviation: NP, nurse practitioner.

^a^ Based on 75 950 practices with tract income data.

^b^ Poverty based on 76 193 practices with tract federal poverty level data.

**Figure 1.**
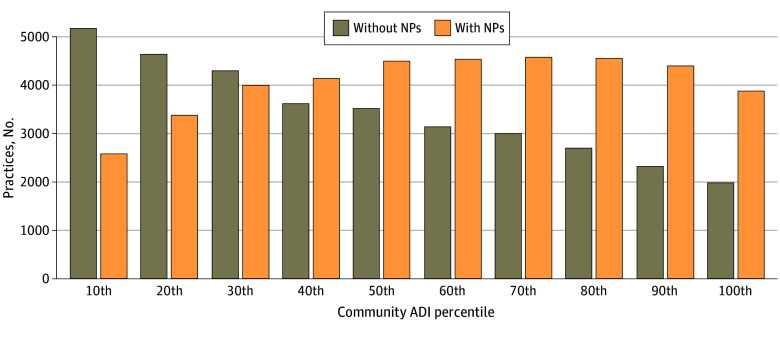
Comparison of the Area Deprivation Index (ADI) Between Communities With Primary Care Practices With or Without Nurse Practitioners (NPs) University of Wisconsin School of Medicine and Public Health ADI is a national percentile ranking with higher numbers referring to a higher level of deprivation. The ADI scores of 74 707 practices (40 382 with NPs) are provided with ADI ranking for their Census Block Group.

In terms of overall socioeconomic disadvantage of the Census Block Group, communities with practices with NPs had significantly higher mean ADI percentiles than communities with practices without NPs (53.3% [95% CI, 53.1%-53.6% vs 42.5% [95% CI, 42.2%-42.7%]; *P* < .001), indicating a greater mean level of socioeconomic disadvantage in communities with practices with NPs. The total number of primary care practices decreased as socioeconomic disadvantage increased, suggesting a potential disparity in access to primary care clinics in areas of higher need (Figure 1). Furthermore, as the number of primary care practices decreased in disadvantaged areas, the proportion of practices with NPs increased. For example, in communities with the lowest decile ADI (ie, least disadvantage), 33.4% of practices employed NPs. In contrast, in communities with the highest ADI (ie, most disadvantaged), 66.0% of practices employed NPs.

**Figure 2.**
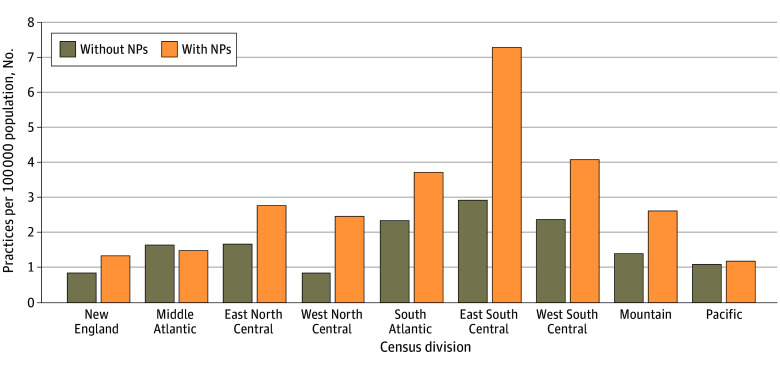
Distribution of the Number of Primary Care Practices in Low-Income Census Tracts With or Without Nurse Practitioners (NPs), Per 100 000 People Across Census Divisions The distribution of practices in low-income Census Tracts is shown across US Census Bureau Census Divisions based on American Community Survey 5-year data (2017-2022) and US 2020 biennial Census 2020 population data of 78 485 practices’ Census Tracts. New England includes CT, MA, ME, NH, RI, and VT. The Middle Atlantic region includes NJ, NY, and PA. The East North Central region includes IL, IN, MI, OH, and WI. The West North Central region includes IA, KS, MN, MO, NE, ND, and SD. The South Atlantic region includes DE, DC, FL, GA, MD, NC, SC, VA, and WV. The East South Central region includes AL, KY, MS, and TN. The West South Central region includes AR, LA, OK, and TX. The Mountain region includes AZ, CO, ID, MT, NV, NM, UT, and WY. The Pacific region includes AK, CA, HI, OR, and WA. A small number of Census Tracts (2%-3%) were missing data, resulting in modestly different sample sizes for each analysis.

Across all US Census Divisions, there were more primary care practices with NPs than without NPs, except for the Middle Atlantic and Pacific Divisions. Similarly, when the analysis was limited to only low-income communities (Figure 2), there were more primary care practices with NPs in low-income communities. This was true in every US Census Division except the Middle Atlantic. In the Middle Atlantic, there were 1.5 practices without NPs for every 1 practice with NPs. In contrast, in low-income communities in the Middle Atlantic, there were almost an equal number of practices with and practices without NPs (1.1 practices without NPs for every 1 practice with NPs). The sensitivity analyses (Table 2) using alternative definitions of *low-income community* and alternative ADI percentiles produced similar results to those presented in Figure 2.

## Discussion

Our analysis offers new insights into the characteristics of communities served by primary care practices employing NPs. First, we found the proportion of primary care practices with NPs in 2023 was 53.4%, which is a substantial increase from prior work that found 21.1% employed NPs in 2012.^20^ Second, we found that primary care practices with NPs were more likely to be located in low-income and rural communities, which is consistent with other studies.^3,20,21,22^ Third, we offer new evidence that primary care practices with NPs, compared with those without NPs, are more likely to serve communities with greater overall socioeconomic disadvantage, lower mean educational attainment, and a greater proportion of residents living below the federal poverty level. Fourth, we show that practices employing NPs predominate in areas with the lowest supply of primary care practices. Fifth, we found in most geographic divisions of the US, there are more primary care practices with NPs than without NPs, a difference that is marked in low-income communities.

These findings on the primary care NP workforce are novel. It is well established that the numbers of NPs are increasing in rural areas, low-income communities, and areas with shortages of health care clinicians.^3,21,22^ However, until now, it was unclear if there were similar patterns for primary care NPs, because most studies do not distinguish between primary and specialty care NPs. This study demonstrates that NPs are increasingly used for primary care delivery across the country, and especially within communities with low socioeconomic status. This finding is important, as fewer medical residents are choosing to practice primary care,^5,23^ resulting in an estimated shortfall of 20 200 to 40 400 primary care physicians by 2036.^24^

### Strengths and Limitations

This study has some strengths, including the use of the IQVIA 2023 OneKey database. Conventionally, it is challenging to identify primary care NPs in large datasets. For example, administrative claims use a single code for all NPs that does not differentiate practice specialty, leading to inaccurate counts.^15,25^ In addition, up to 40% of NP care is hidden in claims because it is billed to a physician (ie, incident to billing).^26^ For these reasons, the OneKey database is a better source than claims for identifying primary care NPs because it relies on a multifaceted approach to identifying primary care practices that includes using facility specialty and predominant physician certification in the practice. It is possible that NPs in these primary care practices provide specialized care, although the extent is unknown.

Another strength is the use of rigorous methods. We conducted several sensitivity analyses to ensure the results were consistent across various definitions of *low-income communities*. We used a robust index to measure overall socioeconomic disadvantage and measured specific socioeconomic characteristics of the community that are associated with health outcomes, such as educational level and economic stability (ie, income, federal poverty status). We examined geographic variation to ensure overall findings were not associated with one specific region. In sum, regardless of the specific measure or geographic unit, the results consistently demonstrate that practices with NPs are more likely to be located in communities with higher levels of socioeconomic disadvantage.

We purposively chose small geographic units of analysis (ie, Census Tracts and Block Groups) because they more accurately capture the socioeconomic position of a community than do zip codes or primary care service areas.^27^ Although these small geographic areas capture the local communities where the practices are located, they may not entirely represent where residents use health care. However, other studies using larger units of analysis (eg, Health Services Area) produced similar findings.^21^

Our analysis also had some limitations. Our dataset included the number of NPs at the practices but no additional information about the number or type of other clinicians or patient volume in the practices was available. We used the most recent RUCA codes, which were last updated in 2010. In addition, we linked zip codes from 2023 OneKey data to the 2021 ADI in the ACS data and there may be some minor misalignment in zip codes year to year.

## Conclusions

The findings of this cross-sectional study carry important implications for researchers and policymakers. Nurse practitioners are the fastest-growing group of health care professionals^1^ and play a key role in increasing access to primary care, especially for at-risk populations.^21,28,29,30^ As the primary care clinician shortage worsens in already underserved communities,^24^ the greater presence of primary care practices with NPs in communities with greater overall socioeconomic disadvantage will be crucial to eliminating disparities in access to primary care. It will be essential to accurately track and project the growing presence of primary care NPs in these areas, as well as their association with population health. It is also important to promote policies, such as strengthening federal and state loan repayment programs, establishing pay parity in state Medicaid programs, and ensuring primary care professional designation for NPs across payers, that help primary care practices attract and retain NPs in underserved areas.^31^ Such steps would expand the capacity of the primary care system to better meet demand in communities where it is needed most.

## Notes

Supplement.
Data Sharing Statement
