# Supplementary material for: Socioeconomic Characteristics of Communities With Primary Care Practices With Nurse Practitioners
Source: JAMA Netw Open. 2025 Feb 28;8(2):e2462360. doi: 10.1001/jamanetworkopen.2024.62360 (PMC11871540; doi:10.1001/jamanetworkopen.2024.62360)
Supplement: Supplement. — Data Sharing Statement [file jamanetwopen-e2462360-s001.pdf]

Data Sharing Statement

O'Reilly-Jacob. Socioeconomic Characteristics of Communities With Primary Care Practices With Nurse Practitioners. *JAMA Netw Open*. Published February 28, 2025. doi:10.1001/jamanetworkopen.2024.62360

Data
Data available: No

Additional Information
Explanation for why data not available: Data is proprietary.
